# Deep learning analysis of left ventricular myocardium in CT angiographic intermediate-degree coronary stenosis improves the diagnostic accuracy for identification of functionally significant stenosis

**DOI:** 10.1007/s00330-018-5822-3

**Published:** 2018-11-12

**Authors:** Robbert W. van Hamersvelt, Majd Zreik, Michiel Voskuil, Max A. Viergever, Ivana Išgum, Tim Leiner

**Affiliations:** 1 Department of Radiology, University Medical Center Utrecht, Utrecht University, P.O. Box 85500, 3508 GA Utrecht, the Netherlands; 2 Image Sciences Institute, University Medical Center Utrecht, Utrecht University, Utrecht, the Netherlands; 3 Department of Cardiology, University Medical Center Utrecht, Utrecht University, Utrecht, the Netherlands

**Keywords:** Artificial intelligence, Myocardial ischemia, Coronary artery disease, Computed tomography angiography

## Abstract

**Objectives:**

To evaluate the added value of deep learning (DL) analysis of the left ventricular myocardium (LVM) in resting coronary CT angiography (CCTA) over determination of coronary degree of stenosis (DS), for identification of patients with functionally significant coronary artery stenosis.

**Methods:**

Patients who underwent CCTA prior to an invasive fractional flow reserve (FFR) measurement were retrospectively selected. Highest DS from CCTA was used to classify patients as having non-significant (≤ 24% DS), intermediate (25–69% DS), or significant stenosis (≥ 70% DS). Patients with intermediate stenosis were referred for fully automatic DL analysis of the LVM. The DL algorithm characterized the LVM, and likely encoded information regarding shape, texture, contrast enhancement, and more. Based on these encodings, features were extracted and patients classified as having a non-significant or significant stenosis. Diagnostic performance of the combined method was evaluated and compared to DS evaluation only. Functionally significant stenosis was defined as FFR ≤ 0.8 or presence of angiographic high-grade stenosis (≥ 90% DS).

**Results:**

The final study population consisted of 126 patients (77% male, 59 ± 9 years). Eighty-one patients (64%) had a functionally significant stenosis. The proposed method resulted in improved discrimination (AUC = 0.76) compared to classification based on DS only (AUC = 0.68). Sensitivity and specificity were 92.6% and 31.1% for DS only (≥ 50% indicating functionally significant stenosis), and 84.6% and 48.4% for the proposed method.

**Conclusion:**

The combination of DS with DL analysis of the LVM in intermediate-degree coronary stenosis may result in improved diagnostic performance for identification of patients with functionally significant coronary artery stenosis.

**Key Points:**

*• Assessment of degree of coronary stenosis on CCTA has consistently high sensitivity and negative predictive value, but has limited specificity for identifying the functional significance of a stenosis.*

*• Deep learning algorithms are able to learn complex patterns and relationships directly from the images without prior specification of which image features represent presence of disease, and thereby may be more sensitive to subtle changes in the LVM caused by functionally significant stenosis.*

*• Addition of deep learning analysis of the left ventricular myocardium to the evaluation of degree of coronary artery stenosis improves diagnostic performance and increases specificity of resting CCTA. This could potentially decrease the number of patients undergoing invasive coronary angiography.*

**Electronic supplementary material:**

The online version of this article (10.1007/s00330-018-5822-3) contains supplementary material, which is available to authorized users.

## Introduction

Assessment of degree of stenosis (DS) in coronary arteries using coronary computed tomography angiography (CCTA) is an accepted diagnostic tool for the detection and exclusion of coronary artery disease (CAD), with consistently high sensitivity and negative predictive value [1–3]. However, it has limited specificity in indicating the functional significance of a stenosis [2, 3]. Invasive fractional flow reserve (FFR) is currently the reference standard to indicate functional significance of a coronary stenosis and to guide treatment [4]. However, due to its invasive nature and high cost, adoption of invasive FFR in clinical practice is limited, and the search for a non-invasive method that would determine functional significance of a stenosis continues.

To address the limited specificity of CCTA, new techniques have been developed to obtain information about the functional significance of a stenosis in a non-invasive way. FFR derived from CT (FFRct) is an emerging method which has shown promising results [5–7]. By simulating flow and pressure through the coronary arteries, a virtual FFR value is obtained. More recently, promising results have been obtained with analysis of myocardial perfusion from resting CT [8–16]. Even though it is well known that perfusion defects are more pronounced under conditions of hyperemia [17, 18], prior studies have shown the feasibility and accuracy of identification of patients with a functionally significant coronary artery stenosis with resting CCTA only [8–13]. With these approaches, functional information is obtained without the need for an additional stress perfusion acquisition, thereby saving radiation and contrast medium dose, lowering risk, and reducing examination duration and cost. In recent studies, approaches exploiting machine learning have been proposed in which the left ventricular myocardium (LVM) in resting CCTA is analyzed and used to classify patients with regard to the presence of functionally significant coronary artery stenosis [8, 9, 13].

In classical machine learning, discriminant features describing the LVM, such as hypo-attenuation and changes in myocardial wall thickness, are manually designed by an expert. Subsequently, these features are used in an algorithm that is built to classify patients according to presence of functionally significant coronary artery stenosis [8, 9]. In contrast to the approaches using expert engineered image features, we recently proposed a deep learning (DL) algorithm, whereby the LVM features that discriminate patients with and without functionally significant coronary artery stenosis are independently learned by the algorithm directly from the image [13]. The current study expands on our previous work [13] by applying a combined method of visual stenosis grading on CCTA and only applying the DL-based analysis to the intermediate-degree stenosis. In contrast to classical machine learning–based approaches, the DL algorithm is able to independently learn generic and complex LVM patterns, and could potentially be more sensitive to changes in the LVM caused by functionally significant stenosis [19, 20].

Thus, the aim of the current study was to evaluate the added value of resting CCTA LVM deep learning analysis over coronary DS evaluation only, for identification of patients with functionally significant coronary artery stenosis.

## Materials and methods

### Study design and population

This was a single center, retrospective, observational study. Between 2012 and 2016, 136 consecutive patients who underwent a CCTA within 1 year prior to an invasive FFR measurement were selected. Patients were excluded (*n* = 10) when automatic LVM segmentation failed or when a significant part was not or incorrectly segmented, following grading defined by Abadi et al [21]. This was the only exclusion criteria used to indirectly exclude scans with bad image quality, suboptimal contrast, motion artifact, or any other artifact. No further in- or exclusion criteria were applied. This study was approved by our local institutional review board at the University Medical Center Utrecht in the Netherlands (protocol number 15/608). The need for informed consent was waived by the institutional review board, since the study was performed on already available imaging data.

### CCTA image acquisition

All patients underwent CCTA on a 256-slice CT system with a collimation of 128 × 0.625 mm (Brilliance iCT, Philips Healthcare). In accordance with the Society of Cardiovascular Computed Tomography guidelines [22], beta-blockers and/or nitroglycerine was administered to target a heart rate of 60 beats per minute (bpm). Patients were imaged using either an ECG-triggered step-and-shoot protocol (≤ 60 bpm) or a retrospectively ECG-gated spiral protocol (heart rate > 60 bpm). An intravenous contrast agent (70–80 mL) (iopromide, Ultravist 300, Bayer Healthcare), followed by a mix (60–67 mL) of 30% contrast agent and 70% saline, followed by saline (30–40 mL) was administrated using a flow rate of 6 (< 80 kg) or 6.7 mL/s (≥ 80 kg). Bolus tracking with 7-s delay was used for acquisition (threshold of 100 HU for step-and-shoot or 200 HU for spiral). For step-and-shoot, images were acquired at 78% of the R-R interval with a rotation time of 0.27 s. A weight-dependent tube voltage and corresponding current of 80, 100, or 120 kVp and 195, 210, and 210 mAs were used in patient weighing < 50 kg, 50–80 kg, and > 80 kg, respectively. For spiral, a heart rate–dependent pitch (0.16–0.18) and rotation time (0.27–0.33) were used. Weight-dependent tube voltage and corresponding tube current were 100, 120, or 120 kVp and 600, 600, and 700 mAs for patients weighing < 65 kg, 65–80 kg, and > 80 kg, respectively. Images acquired at 75% of the R-R interval were selected for further analysis. For both protocols, axial images were reconstructed with a slice thickness of 0.9 mm at 0.45-mm increment using iterative reconstruction (iDose^4^, Philips Healthcare). Maximum DS for each coronary artery was visually scored in consensus by two observers and categorized in 0%, 1–24%, 25–49%, 50–69%, and ≥ 70% or non-diagnostic, in accordance with dedicated guidelines [23, 24].

### Invasive coronary angiography and FFR

**Fig. 1:**
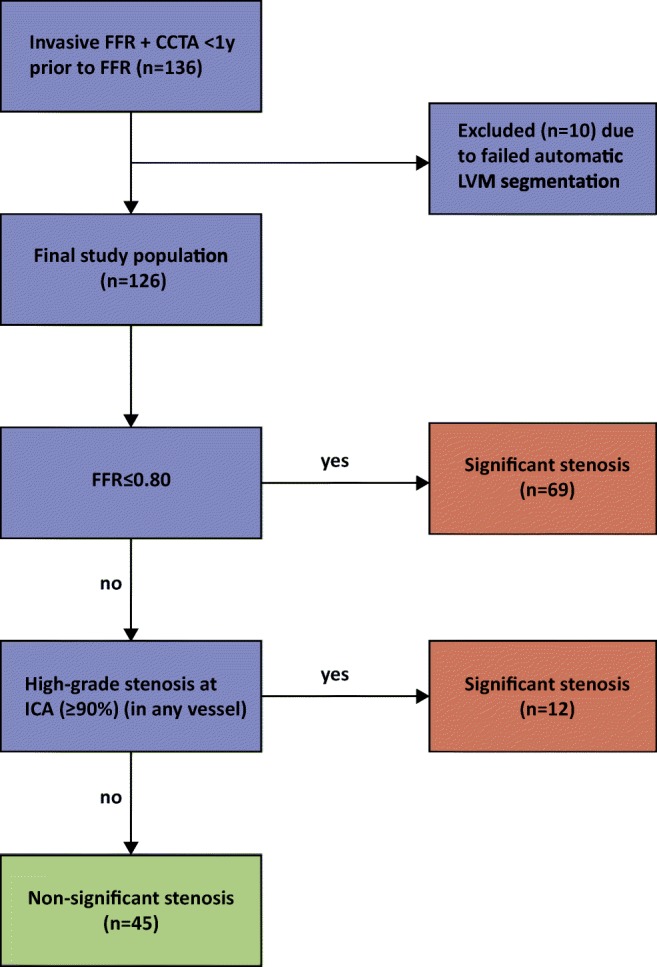
Flowchart indicating ischemia on a patient basis. CCTA, coronary computed tomography angiography; FFR, fractional flow reserve; ICA, invasive coronary angiography; LVM, left ventricular myocardium; n, number of patients

All patients underwent invasive coronary angiography (ICA) and FFR according to standard clinical guidelines. FFR measurements were performed in the coronary arteries when deemed clinically indicated. Measurements were performed under adenosine-induced hyperemia (140 μg/kg as continuous intravenous infusion), and FFR was measured as distal as possible in the target vessel using a coronary pressure wire (Pressure™ Certus™ Wire, St. Jude Medical). The FFR value was automatically calculated by dividing the pressure measured distal to the stenosis by the pressure measured at the level of the guiding catheter in mmHg. In case FFR was ≤ 0.80, the stenosis was considered significant. In the vessels with high-grade coronary stenosis at ICA (≥ 90% DS), invasive FFR was not performed, but stenoses were deemed significant based on DS alone [25]. Therefore, when evaluating ischemia on a patient basis, either a significant FFR (≤ 0.8) or high-grade stenosis at ICA (≥ 90% DS) was used to indicate the presence of functionally significant stenoses (Fig. 1).

### CCTA and deep learning–based image analysis

**Fig. 2:**
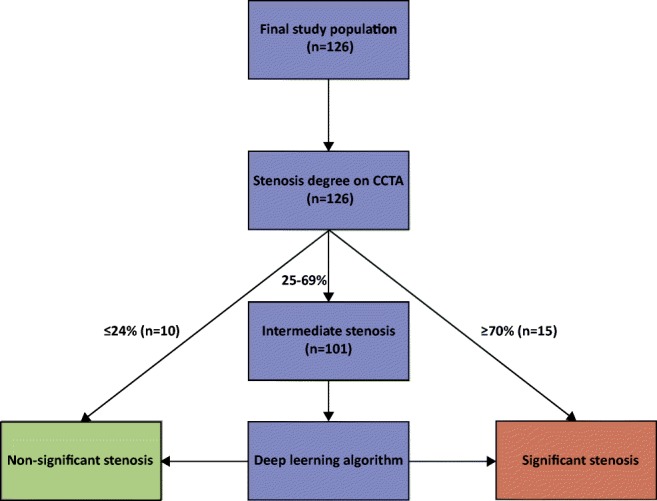
Flowchart of analysis on a patient basis. CCTA, coronary computed tomography angiography; n, number of patients

**Fig. 3:**
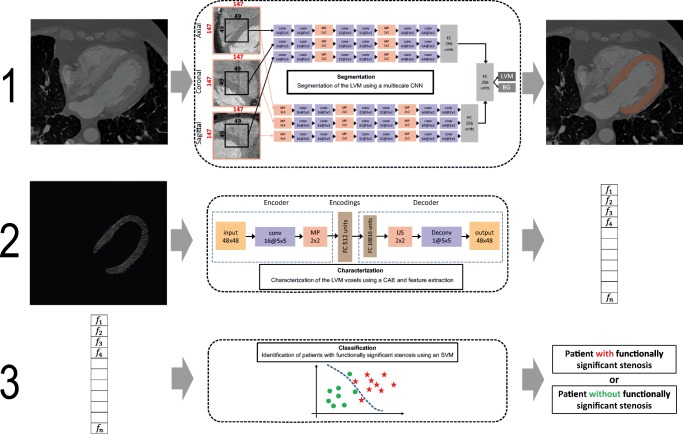
Graphical summary of the utilized deep learning method. The described DL method includes three stages. First (1), LVM is automatically segmented using a multiscale convolutional neural network (CNN). The multiscale CNN includes two identical streams; each analyzes a set of triplanar input patches taken at a single scale (black and red squares) from the axial, coronal, and sagittal image slices with the target voxel in their centers. Each set is processed with a combination of convolutional (conv), max-pooling (MP), and fully connected (FC) layers. In the end, single voxels are classified as myocardium or background. This was performed on all CT slices to segment the whole myocardium. All LVM voxels are analyzed in the next stage. Second (2), LVM was characterized (encoded) on all CT slices by the algorithm in an unsupervised manner using a convolutional auto-encoder (CAE). The CAE contains two parts, an encoder and a decoder. The encoder compresses the data to a lower dimensional representation by convolutional and max-pooling layers. The decoder expands the compressed form to reconstruct the input data by deconvolutional (deconv) and upsampling (US) layers. To represent the entire LVM, statistics over encodings of all LVM voxels (on all CT slices) are used as features. Third (3), based on the extracted features, patients are classified with a support vector machine (SVM) to those with or without functionally significant coronary artery stenosis. BG, background; CAE, convolutional auto-encoder; CCTA, coronary computed tomography angiography; CNN, convolutional neural network; conv, convolutional; deconv, deconvolutional; FC, fully connected; LVM, left ventricular myocardium; MP, max-pooling; SVM, support vector machine; US, upsampling

**Fig. 4:**
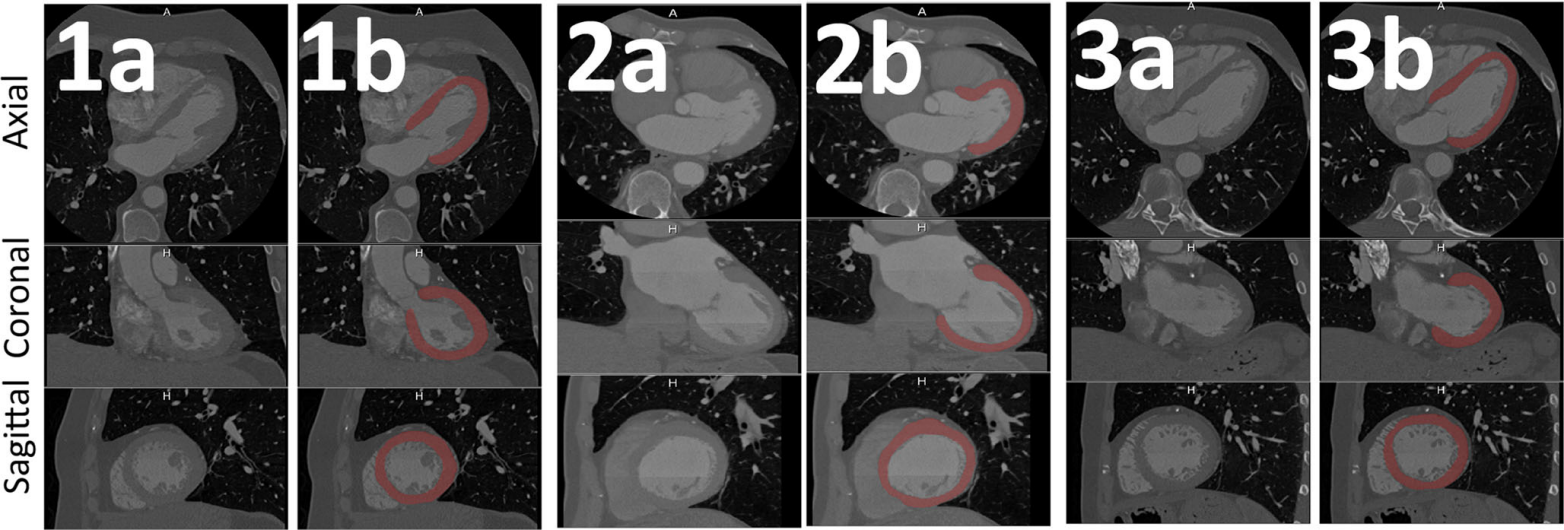
Example of fully automatic left ventricular myocardium segmentation. Coronary CTA images of three randomly selected patients (1, 2, and 3). For each patient, the left column (a) depicts a conventional image, and the right column (b), the fully automatic LVM segmentation overlay. LVM was automatically segmented on all CT slices using a multiscale convolutional neural network. For each patient, an example of one axial slice (top), one coronal slice (middle), and one sagittal slice (bottom) is shown. LVM, left ventricular myocardium

To evaluate the diagnostic performance of adding LVM DL analysis to evaluation of DS, patients were subdivided into three different groups based on the highest degree of coronary stenosis at CCTA (group 1, DS ≤ 24%; group 2, DS 25–69%, and group 3, ≥ 70%DS). Patients with ≤ 24% DS at CCTA (*n* = 10) were considered to have a non-significant stenosis, and patients with ≥ 70% DS at CCTA (*n* = 15) were considered to have a significant stenosis. Patients with intermediate stenosis at CCTA (25–69% DS, *n* = 101) were referred for fully automatic DL analysis of the LVM to indicate whether a functionally significant stenosis was present (Fig. 2). With the DL method, resting CCTA images were automatically analyzed using the method as described by Zreik et al [13]. The prior technical study [13] focused on development of the DL algorithm, while assessing the effect of the different building blocks. The current clinical study expands and validates the previous study in two ways. First, by employing a clinically more relevant reference standard to indicate functional significant stenosis on a patient level: either a significant FFR (≤ 0.8) or high-grade stenosis at ICA ( ≥90% DS). Second, a combined method of visual stenosis grading on CCTA and only applying the DL-based analysis to the intermediate-degree stenosis is applied. In this way, the DL method could be trained and evaluated on this subset of most challenging patient category of intermediate stenosis only. A full description of the DL method can be found elsewhere [13]; a graphical summary is presented in Fig. 3, and a more detailed description about feature extraction in Supplement Fig. 1. First, LVM was automatically segmented on all CT slices using a multiscale convolutional neural network (Fig. 4). Subsequently, the LVM was characterized (encoded) on all CT slices by the algorithm in an unsupervised manner using a convolutional auto-encoder. Using these encodings, which likely contain information regarding shape, texture, contrast enhancement, and more, features were extracted to represent the whole LVM as a volume. Based on these features, patients were classified according to the presence of functionally significant coronary stenosis using a support vector machine classifier. For the current study, the DL method was trained and validated on the subset of patients with intermediate stenosis (*n* = 101) using an invasively measured FFR ≤ 0.80 or angiographic high-grade stenosis (≥ 90% DS) as an indicator for presence of functionally significant stenosis. All patients with intermediate stenosis (*n* = 101) were classified in 10-fold stratified cross-validation experiments. For each 10-fold cross-validation, the dataset was divided into ten stratified subsets whereby one subset was used as validation set and the other nine subsets were used for training. This process was repeated ten times, whereby each subset was used once as validation set. To evaluate robustness of the method, the 10-fold cross-validation was repeated 50 times, with randomization of the data after each repetition. To allow for evaluation of the combined method (DS and LVM DL on intermediate stenosis) on the entire set of patients, patients found to be non-significant (≤ 24% DS) and significant (≥ 70% DS) based on DS on CCTA were assigned probability values of 0 s and 1 s, respectively. For patients with intermediate stenosis, the output probability of the LVM DL model in each cross-validation experiment was employed.

### Statistical analysis

Diagnostic performance of the proposed method (DS evaluation combined with DL analysis of LVM on patients with intermediate stenosis at CCTA) for predicting the presence of functionally significant stenosis on a patient basis was evaluated and compared to the diagnostic performance of DS evaluation alone. Diagnostic performance was evaluated using accuracy, sensitivity, specificity, negative predictive value, positive predictive value, and area under the receiver operating characteristic curve (AUC). For the evaluation of the DS combined with DL analysis, diagnostic performance was evaluated on all 50 cross-validation experiments, and the average ± standard deviation (SD) was used to represent diagnostic performance. For DS only, diagnostic performance is presented with 95% confidence interval (CI). Because prior events and interventions could influence texture changes, an additional sensitivity analysis was performed after exclusion of patients with prior events and interventions (prior myocardial infarction (MI), prior PCI, and/or prior CABG, *n* = 23). The Shapiro-Wilk test was used to evaluate normality of the data. Continuous values are listed as mean with SD and categorical values as percentages, unless stated otherwise. A *p* value < 0.05 was used to indicate statistical significance. IBM SPSS version 21.0 (IBM corp.) and MedCalc Statistical Software version 17.7.2 (MedCalc Software BVBA) were used for statistical analyses.

## Results

### Patient characteristics

In total, 136 patients were eligible, of which 10 were excluded due to incorrect or failed automatic LVM segmentation. The final study population consisted of 126 patients (77% male, mean age 59 ± 9 years). Baseline characteristics are listed in Table 1.

**Table 1:** Patient characteristics

Characteristics	Total population (*n* = 126)	Patients with intermediate stenosis (*n* = 101)
Sex (men), *n* (%)	97 (77)	77 (76)
Age (years), mean ± SD	59 ± 9	60 ± 9
Men	58 ± 9	58 ± 9
Women	65 ± 8	65 ± 8
Body mass index (kg/m^2^), mean ± SD	27 ± 4	28 ± 4
Cardiovascular risk factors, *n* (%)		
Current smoker	31 (25)	23 (23)
Diabetes	24 (19)	21 (21)
Dyslipidemia	109 (87)	87 (86)
Hypertension	110 (87)	89 (88)
Previous myocardial infarction, *n* (%)	14 (11)	11 (11)
Previous percutaneous coronary intervention, *n* (%)	19 (15)	15 (15)
Previous coronary artery bypass grafting, *n* (%)	2 (2)	2 (2)
Coronary calcium Agatston score, median (IQR)	464 (800)^a^	526 (782)^a^

*IQR* interquartile range, *n* number of patients, *SD* standard deviation

^a^in three cases coronary calcium scoring was not performed due to motion artifacts of the coronary arteries

### Invasive coronary angiography and FFR

**Fig. 5:**
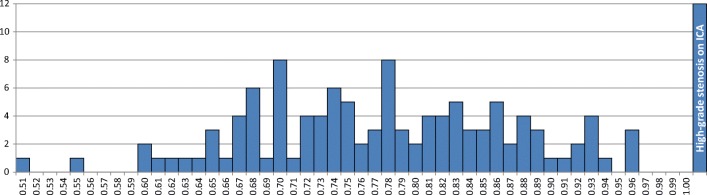
Distribution of lowest FFR value per patient (*n* = 126). Ischemia was evaluated on a patient basis, an FFR ≤ 0.8 or angiographic high-grade stenosis (≥ 90% DS) was used to indicate the presence of a functionally significant stenosis. Per patient, the lowest FFR value is depicted, unless this concerned an FFR > 0.8, and a high-grade stenosis on ICA (≥ 90% DS) was present in another vessel, then this was scored as “high-grade stenosis on ICA”, and FFR value of that patient is not depicted. DS, degree of stenosis; FFR, fractional flow reserve; ICA, invasive coronary angiography

The median number of days (IQR) between CCTA and invasive FFR was 33 (40). Eighty-one patients (81/126, 64%) had a functionally significant stenosis, of which 69 patients (69/81, 85%) had an FFR ≤ 0.80. In the remaining twelve patients (12/81, 15%), lowest FFR value was > 0.80 and a high-grade stenosis on ICA (≥ 90% DS) was present in another coronary artery (Fig. 1). The distribution of severity of disease on a patient-by-patient basis is depicted in Fig. 5.

### Degree of stenosis on CCTA

Maximum DS on a patient basis was 0%, 1–24%, 25–49%, 50–69%, and ≥70% in 2, 8, 10, 91, and 15 patients, respectively. In 20 patients, at least one segment was scored non-diagnostic, of which 18 patients had a significant stenosis (≥ 50% DS) in another segment. In the remaining two patients, maximum DS in another segment was 25–49%, and PCI was present.

### Diagnostic performance

**Fig. 6:**
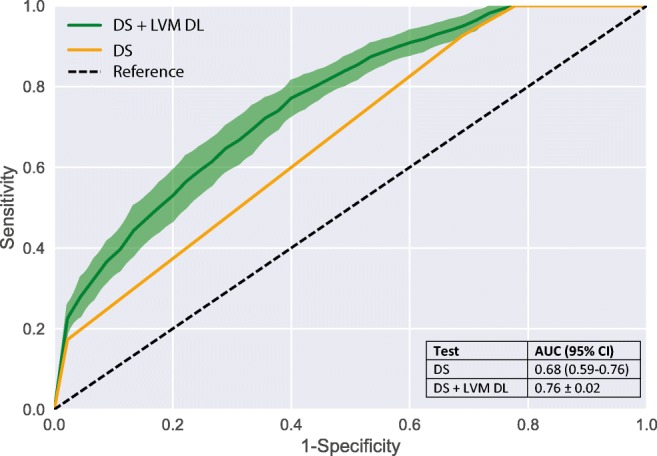
Receiver operating characteristic curves. Diagnostic performance of DS and a combination of DL added to DS on CCTA for predicting functionally significant stenosis on a patient basis. For the combined method, ROC curves and AUC are depicted as average ± SD of 50 cross-validation experiments. AUC, area under the receiver operating characteristic curve; CCTA, coronary computed tomography angiography; DL, deep learning; DS, degree of stenosis; LVM, left ventricular myocardium; ROC, receiver operating characteristic

The diagnostic performance of analysis by evaluation of DS alone to indicate functionally significant stenosis was moderate with an AUC of 0.68 (95%CI 0.59–0.76) (Fig. 6). When applying a threshold of ≥ 50% DS to indicate functional significance of a stenosis, sensitivity was 92.6% (95%CI 84.6–97.2%) and specificity 31.1% (95%CI 18.2–46.6%). Changing the threshold to lower or higher DS resulted in commensurate changes in sensitivity or specificity (Table 2). In ten patients (10/126, 8%), the maximum DS at CCTA was ≤ 24% (Fig. 2). In all of these patients, FFR was higher than 0.8 (mean ± SD; 0.89 ± 0.05) and no high-grade stenosis on ICA was present. Fifteen patients (15/126, 12%) had ≥ 70% DS at CCTA, and in 14 of these patients (14/15, 93%), FFR was ≤ 0.8 or high-grade stenosis on ICA was present. In one case, FFR was 0.82, and no high-grade stenosis on ICA was present. The remaining 101 patients with intermediate DS at CCTA were subjected to LVM DL analysis. Classification based on the combination of DS from CCTA (≤ 24% and ≥ 70%) and LVM DL analysis on intermediate stenosis (25–69%) improved discrimination (AUC = 0.76 ± 0.02) compared to classification based on DS from CCTA only (AUC = 0.68 [95%CI 0.59–0.76]) (Fig. 6). The combined method resulted in an increased specificity of 48.4 ± 0.04% at the expense of a small decrease in sensitivity (84.6 ± 0.03%), compared to the specificity 31.1% (95%CI 18.2–46.6%) and sensitivity 92.6% (95%CI 84.6–97.2%) of DS from CCTA only (≥ 50% DS to indicate functional significant stenosis) (Table 2). To evaluate the robustness of the DL method applied on different patient cohorts, the DL analysis was applied on the complete cohort (*n* = 126) and compared to DL analysis of intermediate stenosis only (*n* = 101) (supplement Fig. 2).

**Table 2:** Diagnostic performance

Method and threshold	Sensitivity (%)	Specificity (%)	PPV (%)	NPV (%)	Accuracy (%)	AUC
CCTA ≥ 25% DS	100.0 (81/81) [95.5–100.0]	22.2 (10/45) [11.2–37.1]	69.8 (81/116) [66.4–73.0]	100.0 (10/10) [100.0–100.0]	72.2 (91/126) [64.3–80.2]	0.68 [0.59–0.76]
CCTA ≥ 50% DS	92.6 (75/81) [84.6–97.2]	31.1 (14/45) [18.2–46.6]	70.8 (75/106) [66.3–74.8]	70.0 (14/20) [49.1–85.0]	70.6 (89/126) [62.6–78.7]	0.68 [0.59–0.76]
CCTA ≥ 70% DS	17.3 (14/81) [9.8–27.3]	97.8 (44/45) [88.2–99.9]	93.3 (14/15) [65.5–99.0]	39.6 (44/111) [37.1–42.3]	46.0 (58/126) [37.2–54.9]	0.68 [0.59–0.76]
CCTA DS + DL combined	84.6 ± 0.03	48.4 ± 0.04	74.7 ± 0.01	64.0 ± 0.05	71.7 ± 0.02	0.76 ± 0.02

Data is given in percentage, data in parentheses is raw data, and data in square brackets is 95% confidence interval. For the combined method, data is depicted as average ± SD of 50 cross-validation experiments. *AUC* area under the receiver operating characteristic curve, *CCTA* coronary computed tomography angiography, *DL* deep learning, *DS* degree of stenosis, *NPV* negative predictive value, *PPV* positive predictive value, *SD* standard deviation

### Diagnostic performance in patients without prior MI, PCI, or CABG (*n* = 103)

In eight patients (8/103, 8%), maximum DS at CCTA was ≤ 24%, and in 12 patients (12/103, 12%), maximum DS was ≥ 70%. DL was applied to the intermediate stenosis (*n* = 83). Also in this sub analysis, the combined method showed an improved discrimination (AUC = 0.77 ± 0.02) compared to evaluation of DS alone (AUC = 0.67 [95%CI 0.57–0.76]) (Supplement Fig. 3). Other measures of diagnostic performance were also comparable to the results of the full sample (Supplement Table 1).

## Discussion

In the current study, we aimed to evaluate the added value of resting CCTA LVM deep learning analysis over coronary DS evaluation only, for identification of patients with functionally significant coronary artery stenosis. We demonstrated that the combination of DS with DL analysis of the LVM in intermediate-degree coronary stenosis improves the specificity of CCTA in comparison to evaluation of the DS alone.

Visual determination of the DS from CCTA is a highly sensitive and established diagnostic tool for evaluation of patients with chest pain of suspected coronary origin. However, CCTA presently lacks specificity for identifying the functional significance of coronary stenosis [1, 2]. A tiered approach with subsequent functional testing (e.g., stress myocardial perfusion imaging) can reduce the number of patients undergoing ICA [26]. However, this approach mandates additional diagnostic testing before deciding on the necessity for PCI. Here, we propose combining evaluation of DS with LVM DL analysis to improve the specificity of CCTA. The combined evaluation has the potential to avoid additional examinations because analysis is performed on the already acquired CCTA images.

Analysis of the LVM on resting CCTA has been previously studied. Two studies evaluated resting dual-energy CT for the detection of perfusion defects confirmed by ≥ 50% stenosis at ICA [15, 16]. They found a slight decrease in sensitivity (79–90%) and increase in specificity (86–92%) compared to ≥ 50% DS on CCTA only (82–98% and 88–91%, respectively). Osawa et al [10] visually evaluated perfusion of the LVM in all cardiac phases from a retrospectively ECG-triggered scan at rest and compared their results with invasive FFR measurements. By combining this LVM analysis with DS, they found an incremental value (AUC = 0.82) over CCTA alone (AUC = 0.71), which is in line with our study. In two recent studies, machine learning–approaches using expert-designed features for classifying patients with functionally significant stenosis were described [8, 9]. Xiong et al [8] found a good discrimination for the detection of a perfusion defect (max. AUC = 0.73). However, in this study, a stenosis degree of ≥ 50% DS on ICA was used as reference [8], and no FFR was performed, making a direct comparison with our results impossible. Han et al [9] used FFR as reference and described that their algorithm, combined with evaluation of DS, showed an added value (AUC = 0.75) over DS alone (AUC = 0.68) [9], which is closely in line with the findings in the present study. However, patients with intermediate DS comprised a minority of the subjects studied by Han et al [9] (33%, 82/252), whereas the present study consisted of a majority of intermediate stenosis (80%, 101/126) and was exclusively focused on the added value of DL in this most challenging patient category.

FFRct is another technique performed on resting CCTA that has shown promising results (AUC = 0.79–0.93) for the evaluation of functionally significant stenosis [5–7]. However, FFRct depends on lumen segmentations which can be challenging or impossible in patients with high-density calcified plaque, motion or misalignment artifacts, and/or prior CABG or PCI [5–7]. Our proposed method may be less affected by these challenges as the analysis of intermediate stenosis is performed only on the LV myocardium. Results of the present study underscore the need to look beyond the coronary arteries in the quest to improve specificity of CCTA. A recent systematic review by Cook et al [27] found that FFRct has a lower diagnostic performance with FFRct values around the cut point (0.7–0.8). In addition, patients with physiologically intermediate lesions (invasive FFR 0.7–0.8) comprised only a small minority of all the patients studied (12.8%) with a median FFR of 0.88, indicating a focus on patients with milder disease. In the current study, nearly three times as many patients (36.5%, 46/126) had physiologically intermediate lesions, and median FFR was 0.78, indicating a more diseased population. This supports the notion that the value of additional analysis of the myocardium is likely to be highest in patients with intermediate stenosis. However, a limitation of the present study is that results are only available on a patient basis; therefore, no indication could be given as to which stenosis was functionally significant, while FFRct has the ability to evaluate functional significance of a stenosis on a vessel basis. It is likely that a combined approach using both techniques will lead to further increase in specificity for identifying flow-limiting lesions in patients with intermediate-degree stenosis at CCTA.

DL algorithms are able to learn new complex patterns and relationships directly from the images without prior specification of which image features represent presence of disease. Therefore, DL may be more sensitive to subtle changes in the LVM caused by functionally significant stenosis, which can be difficult to detect by a human observer [19, 20]. In spite of excellent performance of deep learning techniques demonstrated in many medical image analysis tasks, their interpretability is very limited, and we do not yet fully understand their inner working [19, 28]. Also in this work, even though the extracted encodings are relevant in representing LVM (Supplement Fig. 1), they are not readily interpretable. As distinct combinations of encodings represent different LVM appearances (e.g., normal vs. thin), specific encodings do not correspond to specific physical appearances. This prevented us from interpreting, visualizing, and localizing differences within the LVM for patients with significant stenosis. Future work might address these limitations [19, 28].

This study has limitations. First, the retrospective single-center study design has to be taken into account. This could have induced selection bias as CT could have been the reason for referral to invasive FFR. In addition, because of this bias, the study group consisted of patients with high Agatston scores and a high prevalence of stenosis and extensive CAD with mainly intermediate stenosis (*n* = 101/126). Due to blooming, calcified plaques are known to cause overestimation of DS and thereby decrease specificity [29]. In addition, as reported in prior studies [2, 3], DS on CCTA showed high diagnostic accuracy for low- and high-grade stenosis (≤ 24% and ≥ 70%), which decreased when evaluating intermediate-degree stenosis (25–69%).This is reflected in the low specificity for DS only on CCTA found in the current study (31.1%) compared to reported in literature (40–83%) [1–3]. Second, maximum DS for each coronary artery was visually categorized, and no continuous quantitative measurements were performed. Although continuous quantitative measurements would allow for a more accurate way to evaluate diagnostic accuracy of DS, this is not regularly performed in clinical practice and was therefore not performed. Third, in the current study, no comparison with another type of functional assessment was performed (e.g., FFRct or visual assessment of myocardial perfusion), thereby limiting the evaluation of the added value of the current method compared to other available methods. Fourth, we used equipment of one single vendor and results may therefore be limited to this vendor. A final limitation is the relative small patient cohort and unbalanced dataset, which can introduce bias in performance. Although we performed 50 repetitions of 10-fold cross-validation experiments with randomization after each repetition, results may still be partially affected by coincidental findings. Analysis on a separate patient cohort as well as prospective studies needs to be performed to assess whether the found correlation also implies causation. Future work will address these limitations.

In conclusion, combining assessment of degree of stenosis with DL analysis of the LVM may result in improved diagnostic performance for identification of patients with functionally significant coronary artery stenosis. Future research is warranted to evaluate this approach on patients with low pre-test probability of obstructive coronary disease, more clinically encountered at CCTA.

## Electronic supplementary material

Supplemental Figure 1 (DOCX 590 kb)

Supplemental Figure 2 (DOCX 181 kb)

Supplemental Figure 3 (DOCX 116 kb)

Supplemental Table 1 (DOCX 14 kb)

## Abbreviations

AUC: Area under the receiver operating characteristic curve
CABG: Coronary artery bypass grafting
CAD: Coronary artery disease
CCTA: Coronary computed tomography angiography
DL: Deep learning
DS: Degree of stenosis
FFR: Fractional flow reserve
ICA: Invasive coronary angiography
LVM: Left ventricular myocardium
PCI: Percutaneous coronary intervention
