# Supplementary material for: Deep learning analysis of left ventricular myocardium in CT angiographic intermediate-degree coronary stenosis improves the diagnostic accuracy for identification of functionally significant stenosis
Source: Eur Radiol. 2018 Nov 12;29(5):2350–9. doi: 10.1007/s00330-018-5822-3 (PMC6443613; doi:10.1007/s00330-018-5822-3)
Supplement: Supplementary file 1 — (DOCX 590 kb) [file 330_2018_5822_MOESM1_ESM.docx]

**Supplement Figure 1. Encoding the LVM in the DL method using a convolutional auto-encoder.** Because the discriminative features of the LVM for functionally significant stenosis on CCTA at rest are not well-defined, we applied an unsupervised approach using a convolutional auto-encoder (CAE). Unlike most studies, where features were designed by hand, in the current study features were based on the encodings extracted in an unsupervised fashion by the DL method using a CAE. To ensure that these encodings represent the myocardium and not another random organ or structure, only small local image patches of the segmented myocardium (A) were introduced to the CAE and thereafter encoded (B). All voxels of the segmented myocardium in all slices were encoded by the CAE using patches around them, as illustrated by an example patch (blue box) in A. To ensure that the extracted encodings would be relevant in representing the myocardial patches, the CAE was trained by forcing it to reconstruct (decode) the input myocardium patches using only these encodings, as illustrated by three randomly selected patches in B. To train the CAE, the input myocardial patches were compressed (encoded) to N=512 values (encodings) and reconstructed (decoded) back. The mean squared error between the input patches and the reconstructed patches was computed during training and minimized iteratively. This minimization forced the encodings to contain relevant information; successfully representing the myocardium appearance.
