# Supplementary material for: Deep learning analysis of left ventricular myocardium in CT angiographic intermediate-degree coronary stenosis improves the diagnostic accuracy for identification of functionally significant stenosis
Source: Eur Radiol. 2018 Nov 12;29(5):2350–9. doi: 10.1007/s00330-018-5822-3 (PMC6443613; doi:10.1007/s00330-018-5822-3)
Supplement: Supplementary file 2 — (DOCX 181 kb) [file 330_2018_5822_MOESM2_ESM.docx]

**Supplement Figure 2. Receiver operating characteristic curves of DL applied to all patients (A, n=126) and patients with intermediate stenosis only (B, n=101).** To evaluate robustness of the DL analysis on low- or high-grade stenosis, we also evaluated the performance of our DL algorithm on the complete cohort (n=126). We retrained and evaluated the DL method on all 126 cases using the same scheme of 10-fold cross-validation with 50 repetitions. The resulting ROC curve and AUC are shown in A. These results are comparable with the performance of DL on the intermediate stenosis only (n=101), used in the current study and shown in B. This indicates that comparable discriminative power (but also comparable misclassification) are achieved by the DL algorithm, regardless of the population. However, it should be noted that the entire dataset is obtained from a heavily diseased patient cohort, and thus none of these patients are considered as an “easy case” for classification. *AUC = area under the receiver operating characteristic curve; DL = deep learning; LVM = left ventricular myocardium; ROC = receiver operating characteristic*
