# Supplementary material for: Deep learning analysis of left ventricular myocardium in CT angiographic intermediate-degree coronary stenosis improves the diagnostic accuracy for identification of functionally significant stenosis
Source: Eur Radiol. 2018 Nov 12;29(5):2350–9. doi: 10.1007/s00330-018-5822-3 (PMC6443613; doi:10.1007/s00330-018-5822-3)
Supplement: Supplementary file 3 — (DOCX 116 kb) [file 330_2018_5822_MOESM3_ESM.docx]

**

**Supplement Figure 3. Receiver operating characteristic curves for patients without prior MI, CABG and/or PCI (n=103).** Diagnostic performance of DS and a combination of DL added to DS from CCTA for predicting functionally significant stenosis on a patient basis. For the combined method, ROC-curves and AUC are depicted as average ± SD of 50 cross-validation experiments. *AUC = area under the receiver operating characteristic curve; CABG = coronary artery bypass grafting; CCTA = coronary computed tomography angiography; DL = deep learning; DS = degree of stenosis; LVM = left ventricular myocardium; MI = myocardial infarction; PCI = percutaneous coronary intervention; ROC = receiver operating characteristic*
