# Supplementary material for: Deep learning analysis of left ventricular myocardium in CT angiographic intermediate-degree coronary stenosis improves the diagnostic accuracy for identification of functionally significant stenosis
Source: Eur Radiol. 2018 Nov 12;29(5):2350–9. doi: 10.1007/s00330-018-5822-3 (PMC6443613; doi:10.1007/s00330-018-5822-3)
Supplement: Supplementary file 4 — (DOCX 14 kb) [file 330_2018_5822_MOESM4_ESM.docx]

**Supplement Table 1. Diagnostic performance for patients without prior MI, CABG and/or PCI (n=103).**

| **Method and threshold** | **Sensitivity (%)** | **Specificity (%)** | **PPV (%)** | **NPV (%)** | **Accuracy (%)** | **AUC** |
| --- | --- | --- | --- | --- | --- | --- |
| **CCTA ≥25% DS** | 100.0 (67/67) [94.6-100.0] | 22.2 (8/36) [10.1-39.2] | 70.5 (67/95) [66.8-74.0] | 100.0 (8/8) [100.0-100.0] | 72.8 (75/103) [64.1-81.6] | 0.67 [0.57-0.76] |
| **CCTA ≥50% DS** | 92.5 (62/67) [83.4-97.5] | 30.6 (11/36) [16.3-48.1] | 71.3 (62/87) [66.4-75.7] | 68.8 (11/16) [45.3-85.4) | 70.9 (73/103) [62.0-79.8] | 0.67 [0.57-0.76] |
| **CCTA ≥70% DS** | 16.4 (11/67) [8.5-27.5] | 97.2 (35/36) [85.5-99.9] | 91.7 (11/12) [59.7-98.8] | 38.5 (35/91) [35.7-41.3] | 44.7 (46/103) [34.9-54.4] | 0.67 [0.57-0.76] |
| **CCTA DS + DL combined** | 84.8 ± 0.04 | 50.5 ± 0.04 | 76.1 ± 0.02 | 64.5 ± 0.05 | 72.8 ± 0.02 | 0.77 ± 0.02 |

Data is given in percentage, data in parentheses is raw data and data in brackets is 95% confidence interval. For the combined method, data is depicted as average ± SD of 50 cross-validation experiments. *AUC = Area under the receiver operating characteristic curve, CABG = coronary artery bypass grafting; CCTA = coronary computed tomography angiography; DL = deep learning; DS = degree of stenosis;* *LVM = left ventricular myocardium; MI = myocardial infarction; NPV = negative predictive value; PCI = percutaneous coronary intervention; PPV = positive predictive value; SD = standard deviation*
